# Effect of Er:YAG Laser for Women with Stress Urinary Incontinence

**DOI:** 10.1155/2019/7915813

**Published:** 2019-01-15

**Authors:** Kun-Ling Lin, Shih-Hsiang Chou, Cheng-Yu Long

**Affiliations:** ^1^Department of Obstetrics and Gynecology, Kaohsiung Medical University Hospital, Kaohsiung Medical University, Kaohsiung, Taiwan; ^2^Graduate Institute of Clinical Medicine, College of Medicine, Kaohsiung Medical University, Kaohsiung, Taiwan; ^3^Department of Orthopedics, Kaohsiung Medical University Hospital, Kaohsiung Medical University, Kaohsiung, Taiwan

## Abstract

**Purpose:**

The aim of our study is to assess efficacy of noninvasive erbium-doped yttrium aluminium garnet laser (Er:YAG laser) for female stress urinary incontinence (SUI).

**Materials and Methods:**

Forty-one women with SUI were included in the study and scheduled for vaginal Er:YAG laser treatment. The procedure was performed with a 2940 nm, Er:YAG laser (Fotona Smooth ™ XS, Fotona, Ljubljana, Slovenia), designed to heat up the vaginal mucosa to around 60°C. All subjects had a baseline and 6 months' posttreatment assessment that included perineal sonography and lower urinary tract symptoms.

**Results:**

Significant improvements in both urinary frequency and incontinence were found 6 months after Er:YAG laser treatment when compared to the baseline results (p<0.001). The battery of questionnaires administered to patients, including the UDI-6, IIQ-7, OABSS, and POPDI-6, all showed significant improvement posttreatment (P < 0.001). The treatment efficacy for the vaginal Er:YAG laser for SUI at 6 months posttreatment was 75.5% (31/41). Bladder neck mobility by perineal ultrasonography decreased significantly (16.1 ± 6.4 mm to 10.5 ± 4.6 mm) after treatment (p=0.039). No permanent adverse events were found.

**Conclusions:**

The Er:YAG vaginal laser seems to be a safe and efficacious treatment for women with mild to moderate SUI, this being partly related to the decrease of bladder neck mobility following laser treatment.

## 1. Introduction

Female urinary incontinence means involuntary urine leakage. Approximately 4%–35% of adult women suffer from stress urinary incontinence, defined as an involuntary leakage on effort or exertion, or on sneezing or coughing [1]. Stress urinary incontinence (SUI) is a significant public health problem, and a variety of risk factors have been investigated, including pregnancy, modes of delivery, aging, obesity, and smoking [2]. During pregnancy, 31%–47% of pregnant women experience an episode of SUI and 34% of women still had SUI postpartum [3–5]. Nearly 73% of women assessed with SUI three months postpartum were also found to have persistent SUI six years postpartum [2, 6–8]. Therefore, more researches into current therapies used to treat SUI have been investigated to improve patients' quality of life.

The treatment of SUI includes conservative treatment and surgical intervention. The conservative treatment methods focus on lifestyle modification, bladder and pelvic muscle training, and electronic stimulation of pelvic floor muscles. Although conservative treatment can attain excellent results, many patients experience poor outcomes due to low persistence and compliance rates [9, 10]. Regarding surgical intervention for SUI, the middle urethral sling procedure is the gold standard surgery and is regarded as a highly effective treatment [11, 12].

However, the adverse effects of transvaginal sling surgery may occur, including mesh extrusion, voiding dysfunction, dyspareunia, and pain. Due to these possible complications, patients are often reluctant to undergo surgical intervention [13, 14]. Furthermore, the American Food and Drug Administration (FDA) has announced warnings regarding their use. Therefore, the risks of these devices should be evaluated, and patients should be informed prior to use [15].

In 2014, the FDA approved the indication of the noninvasive, Er:YAG laser in the field of urogynecology. The Er:YAG laser has a SMOOTH mode, which emits laser pulses from the vaginal probe, releasing pulsatile-heat to the vaginal wall to shorten the intermolecular cross- links of collagen, shrinking the collagen fibril, and enhancing collagen production [16, 17]. The thermal effect of the vaginal laser seems to strengthen the support of the vaginal wall via neocollagenesis and subsequently helps to treat SUI. Our study aims to assess the clinical outcome of this new noninvasive Er:YAG laser treatment in women with SUI.

## 2. Material and Methods

A retrospective study was conducted by the Department of Obstetrics and Gynecology, Kaohsiung Medical University Hospital, Taiwan. The study protocol was approved by the Institutional Review Board of Kaohsiung Medical University Chung-Ho Memorial Hospital Research (IRB No:KMUHIRB-E(I)- 20180109). It included 100 patients suffering from SUI between August 2015 and December 2016. These women were classified using the Ingelman-Sundberg method of stress incontinence classification [18]. They were classified into three grades based upon their clinical severity. Grade I: urinary incontinence when coughing or sneezing. Grade II: urinary incontinence when running or lifting objects off the floor. Grade III: urinary incontinence when walking or climbing stairs. The grades of Ingelman-Sundberg are in accordance with Stamey as grade I:mild (SUI only with severe stress, such as coughing or sneezing), grade II:moderate (incontinence with minimal stress, including walking or running), and grade III:severe (incontinence at bed rest) [19]. All patients underwent three treatments, using the Er-YAG vaginal laser (Fotona Smooth ™ XS, Fotona, Ljubljana, Slovenia) with a wavelength of 2940 nm, every 4 weeks.

We performed the vaginal laser procedures according the manufacturer's guides. During treatment, the patient was set in lithotomy position without any anesthesia usage. After sterile procedures, the speculum and laser delivery handpiece were inserted into the vaginal canal. The treatment protocol consisted of three steps. In the first step, the circular vagina, from the distal point to the introitus, was irradiated with a full spot R11 handpiece. Three passes were performed with a fluence of 10 J/cm^2^ and 1.6 Hz using smooth mode. In the second step, a fractional laser beam was emitted perpendicularly to the anterior wall using a PS03 handpiece with fluence of 10 J/cm^2^ and 1.6 Hz with smooth mode for 5 passes. Finally, the introitus os and vestibule were irradiated with a shooting pattern using a PS03 handpiece for 3 passes.

Before and 6 months after treatment, each patient's baseline characteristic data was collected and a personal interview was conducted using the following battery of questionnaires: Incontinence Questionnaire–Short Form (ICIQ-SF) [20], Urogenital Distress Inventory 6 (UDI-6), Incontinence Impact Questionnaire 7 (IIQ-7) [21], Overactive Bladder Symptom Score (OABSS) [22], and Pelvic Organ Prolapse Distress Inventory 6 (POPDI-6) [23]. The information gained was used to assess each patient's degree of urinary incontinence and its impact on their quality of life.

**Figure 1:**
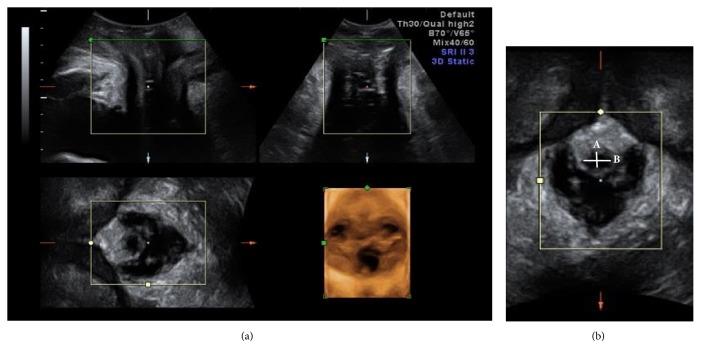
(a)** Perineal sonography**. Lower urinary tract on three-dimensional transperineal ultrasound imaging in a woman. The top left image shows the mid-sagittal view (A-plane). The top right image is a coronal view (B-plane); the bottom left is the axial or C-plane, showing hypoechogenic area of urethra. The bottom right image is the resulting rendered volume. (b)** Axial view of the urethral hypoechoic core**. Ultrasound image (axial plane) of the mid-urethral hypoechoic core in a woman with SUI, showing the measurements made for this study: the shortest (A) and longest (B) diameters of the urethral hypoechoic core.

**Figure 2:**
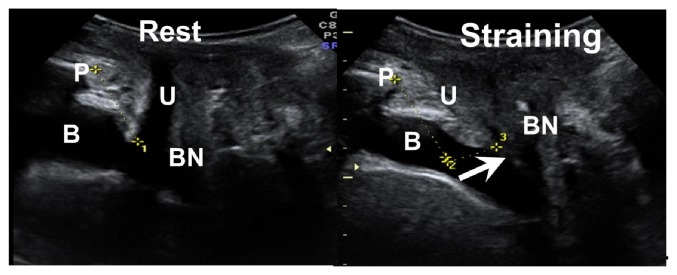
The sagittal view of perineal sonography. The pubic bone marked as P. The urethra marked as U. The bladder marked as B. The bladder neck marked as BN. The pubic bone is set as basic point and makes a line from the pubic bone to BN at rest and during straining. The maximal distance of two lines is calculated as bladder mobility.

Perineal ultrasound was performed using the Volusion General Electric Sonography, expert 730 type (GE, Healthcare Ultrasound, Zipf, Austria), with 3.5 MHz curved linear-array. The transducer was placed between the major labia and underneath the external urethral orifice. The assessments included calculation of the hypoechoic area of the proximal, middle, and distal urethra by multiplying *π* by the lengths of the short (A) and long (B) axes of the urethral core at rest and during Valsalva maneuver using three-dimensional mode (Figure 1). The sagittal view was used to measure bladder neck mobility at rest and during Valsalva maneuver at baseline and three months after treatment (Figure 2).

The vaginal pressure was measured at rest and during contraction prior to initial treatment and repeated 6 months after laser treatment. The average and maximal pressures and the period of time during contraction were also calculated. If patients did not adhere to their scheduled follow-up appointments, their recordings were excluded from the study. For normal distribution, data was reported as mean and standard deviations. Statistical significance was set to p < 0.05. Confidence intervals were set at 95%. Two-way analysis of variance for repeated measures and factorial analysis of variance were used to test the differences within and between the groups.

The ethics committee of our university hospital approved the study protocol. (No.: KMUHIRB- E(I)-20180109)

## 3. Results

All 41 patients completed the study. The demographic characteristics of the patients are presented in Table 1. The mean age of the patients was 45.9 ± 7.2 years. Thirty-three women were menopausal. The median parities were 2.1 ± 0.8. The distribution among the grade levels of severity of SUI is as follows: grade I, 8 patients; grade II, 24 patients; and grade III, 4 patients.

The ICIQ-SF median score, which assessed the frequency and severity of incontinence, before treatment was 7.2 ± 4.5 and significantly decreased to 3.7 ± 3.5 posttreatment (p<0.001) (Table 2). The UDI-6 and IID-7 were used to assess the symptoms of distress and the impact of urinary incontinence on the subjects' daily life; both were significantly decreased after treatment (p < 0.01). The OABSS significantly improved with scores from 4.6 ± 3.0 to 2.2 ± 2.2 (p=0.001). The scores of POPDI-6, which measured distress and discomfort of pelvic organ prolapse or vaginal relaxation, were significantly decreased from 5.03 ± 4.5 to 1.0 ± 1.9 (p< 0.001). At 6 months after treatment, 36.6% (15/41) of patients were cured of SUI, with 39% (16/41) of patients reporting improvement. In ten patients (24.4%), there was no improvement or worsening of their SUI symptoms. The treatment efficacy of Er:YAG laser 6 months posttreatment was 75.5% (31/41) (Table 3).

After Er:YAG laser treatment, measurements of the middle urethral short axes (A), long axes (B), and middle urethra areas showed an obvious decrease at rest (A: 54 ± 1.2 mm to 4.7 ± 1.0 mm, p=0.033; B: 7.4 ± 2.1 mm to 6.4 ± 1.7 mm, p=0.024; area: 130 ± 58 mm^2^ to 93.5 ± 42.7 mm^2^, p=0.001). The proximal urethral short axes (A) and the distal urethral long axes (B) were also significantly decreased (A: 5.6 ± 1.9 mm, p=0.028; B: 7.0 ± 2.0, p= 0.001) (Table 4). During straining, the measurements of the mid-urethral areas and short axes (A) were significantly decreased (area: 90.0 ± 57.4mm to 80.0 ± 30.9 mm, p=0.048; A: 2.2 ± 3.3 mm to 2.3 ± 3.4, p=0.006) (Table 5). Bladder neck mobility from resting to straining via perineal sonography showed significant decrease after laser treatment (16.1 ± 6.4 mm to 10.5 ± 4.6 mm, p=0.039) (Figure 2).

The measurement of the strength of voluntary contractions of the pelvic floor muscles (perineometry) was evaluated using vaginal air pressure (Table 6). There was no significant difference in vaginal air pressure at rest or during contraction after treatment. The maximal and average vaginal pressure readings obtained during contraction were not significantly increased, and neither was the contraction time after treatment with the Er:YAG vaginal laser. The results were not significant.

## 4. Discussion

Initially, our study included 100 patients, but ultimately only 41 patients completed the course. The major reason for exclusion was due to rapid resolution of symptoms after one or two treatments with the Er:YAG vaginal laser and subsequent patient self-discontinuation of follow-up treatment. As previous studies have reported, there are no long-term complications when using minimally invasive, nonsurgical, or nonablative laser therapy [12, 16, 17]. The most commonly reported adverse effects from the Er:YAG vaginal laser, in our study, were a burning sensation during the procedure and less vaginal bleeding during the first week posttreatment. Even FDA reminded the application of vaginal laser but the Er:YAG vaginal laser is not included in the warning list.

The efficacy of our treatment using the Er:YAG laser was approximately 75.5%, and is comparable to the efficacy results reported in the study by Tien YW et al. [24]. The 90% of women who underwent the Er:YAG vaginal laser treatment in our study were classified as either grade I or grade II SUI; therefore the efficacy for treatment for severe SUI (grade III) should be further investigated. In other studies, patient responses were obtained using associated incontinence questionnaires (ICIQ score) and showed a significant increase from 2.5 to 8 points after Er:YAG laser treatment [25, 26]. We obtained similar results that the ICIQ questionnaire results showed a significant improvement with an initial score of 7.2 ± 4.5 down to posttreatment score of 3.7 ± 3.5.

As previous studies have indicated, the patients' quality of life and symptoms of incontinence, assessed using UDI-6 and IID-7, improved after treatment with the Er:YAG laser [24, 26]. But we also have noticed that the pure cure-rate is not as high as expected with 36.6% of women still suffering from persistent SUI. In comparison to other studies, our OABSS and POPDI scores were significantly decreased. Women with SUI usually have OAB problem and most parous women demonstrated some degree of cystocele. The mechanism has been investigated that funneling of the proximal urethra urine enters the proximal urethra and then produces sensory stimulation resulting in a reflex of bladder contraction with OAB [27]. The Er:YAG vaginal laser treatment has proven to have a supportive effect on the bladder; therefore we expected that it could also improve symptoms of SUI, then with relatively less bladder contractions to improve OAB and also less prolapse associated disturbance. From our ultrasound data, we further demonstrated the supportive effect of Er:YAG vaginal laser treatment on the bladder and urethra, as evidenced by a decrease in measured bladder neck mobility (16.1 ± 6.4 mm to 10.5 ± 4.6 mm). Chen GD et al. reported that a woman with the bladder neck decent during straining over 13 mm is a predictor of SUI [28]. The change in bladder neck mobility after Er:YAG laser treatment explains not only the positive effect on bladder stability, but also the improvement of SUI symptoms in our study.

Due to SUI often being related to pelvic floor muscle dysfunction, we were inquisitive about a possible application using the Er:YAG laser to increase pelvic muscle tone [29, 30]. Disappointingly, the assessed muscle tone did not show a significant increase after treatment, but other improvement trends found in our study were corroborated by a previous study [31]. We took into consideration that the wave emitted by the Er:YAG vaginal laser could only penetrate around 1-2 mm, so the laser's effectiveness on increasing pelvic muscle tone would be decreased [32]. On the other hand, a synergistic benefit may be achieved with a combined treatment regimen using the Er:YAG laser in combination with pelvic muscle exercises, thereby allowing for a different mechanism for SUI improvement.

In the previous study by Petros PE et al. and Long et al. [33, 34], measurements were taken of the urethral hypoechoic area of the proximal, middle, and distal urethra via perineal sonography before and after performing sling surgery, while at rest and straining. As expected, the middle urethral hypoechoic area was significantly decreased at rest and during straining after Er:YAG laser treatment. The results obtained seem to provide proof that the mechanism of incontinence is located at the middle urethra, according to the integral theory. The decreased urethral hypoechoic area effect was more evident at rest than during straining. We infer that the bladder support change after Er:YAG vaginal laser treatment may allow remodeling of the lower urinary tract anatomy due to the mechanical traction of thermal effect and will thereby further improve SUI. The limitations of our study are the small sample size due to difficulty to return for finishing three times of treatment and possible recall bias with questionnaires. Regarding symptom evaluation, perineal sonography has been applied; however, assessment should include pad test and urodynamic study for more objective results.

## 5. Conclusion

Er:YAG vaginal laser is an effective treatment for women with mild to moderate SUI. No severe or permanent side effects occurred during or after treatment. The bladder neck mobility from perineal sonography was significantly decreased after Er:YAG vaginal laser treatment, which contributed to the improvement of SUI symptoms. More prospective studies and longer follow-up would be helpful to confirm our findings and realize the appropriate interval for repeat intervention.

## Figures and Tables

**Table 1:** Demographic data (n=41) are given as mean ± standard deviation or n(%).

Mean age (years)	45.9 ± 7.2
Mean parity	2.1 ± 0.8
Mean BMI (kg/m2)	22.8 ± 3.6
Maximal birth weight (kg)	3.4 ± 0.3
Pad test	5.3 ± 2.1
Menopause	13 (24.5)

SUI grade	
grade 1	8 (19.5)
grade 2	24 (70.7)
grade 3	4 (9.7%)
Follow up (months)	6

BMI, body mass index.

Values are expressed as mean ± standard deviation or numbers.

**Table 2:** Questionnaires from baseline before the intervention and 6 months posttreatment.

n=41	Baseline	6 months post laser	P value*∗*
ICIQ-SF	7.2 ± 4.5	3.7 ± 3.5	<0.001*∗*
UDI-6	32.9 ± 18.3	15.9 ± 15.4	<0.001*∗*
IID-7	25.2 ± 21.5	14.4 ± 19.2	<0.001*∗*
OABSS	4.6 ± 3.0	2.2 ± 2.2	0.001*∗*
POPDI-6	5.03 ± 4.5	1.0 ± 1.9	<0.001*∗*

Values are expressed as mean ± standard deviation or numbers.

*∗*Statistical significance; paired t-test.

**Table 3:** Efficacy of Er-YAG laser.

N=41	6 months post treatment: n(%)
Cure	15 (36.6)
Improved	16 (39.0)
Failure	10 (24.4)
Efficacy of laser	31/41 (75.5%)

**Table 4:** Resting urethral topography at baseline and 6 months after treatment.

Rest	Baseline	6 months post laser	P-value*∗*
Proximal (area)(mm^2^)	122.3 ± 54.2	95 ± 52.4	0.099
A (mm)	5.6 ± 1.9	4.7 ± 1.5	0.028*∗*
B (mm)	6.8 ± 1.7	6.1 ± 1.5	0.965
Middle (area)(mm^2^)	130.6 ± 58	93.5 ± 42.7	0.001*∗*
A (mm)	5.4 ± 1.2	4.7 ± 1.0	0.033*∗*
B (mm)	7.4 ± 2.1	6.4 ± 1.7	0.024*∗*
Distal (area)(mm^2^)	107.2 ± 52.2	78.9 ± 61.8	0.003*∗*
A (mm)	4.7 ± 1.2	4.0 ± 1.2	0.152
B (mm)	7.0 ± 2.0	5.8 ± 2.0	0.001*∗*

Values are expressed as mean ± standard deviation or numbers.

*∗*Statistical significance; paired t-test.

**Table 5:** Straning urethral topography at baseline and 6 months after treatment.

Straining	Baseline	6 months post laser	P-value*∗*
Proximal (area)(mm^2^)	102.4 ± 47.1	95.0 ± 55.9	0.553
A (mm)	4.8 ± 1.3	4.0 ± 3.8	0.319
B (mm)	6.5 ± 1.6	5.5 ± 4.7	0.208
Middle (area)(mm^2^)	90.0 ± 57.4	80.0 ± 30.9	0.048*∗*
A (mm)	2.2 ± 3.3	2.3 ± 3.4	0.006*∗*
B (mm)	7.6 ± 4.2	6.5 ± 4.3	0.065
Distal (area)(mm^2^)	76.0 ± 59.6	78.0 ± 44.1	0.001*∗*
A (mm)	2.5 ± 3.7	1.2 ± 2.6	0.137
B (mm)	5.5 ± 4.7	6.4 ± 4.7	0.301
Bladder neck mobility (mm)	16.1 ± 6.4	10.5 ± 4.6	0.039*∗*

Values are expressed as mean ± standard deviation or numbers.

*∗*Statistical significance; paired t-test.

**Table 6:** Perineometry at baseline and 6months after treatment.

Perineometry	Baseline (n = 41)	6-month follow-up (n = 31)	P value*∗*
Resting (mmHg)	32.6 ± 12.6	35 ± 19.0	0.136
Contraction			
Maximal (mmHg)	58.6 ± 21	60.3 ± 25.5	0.691
Average (mmHg)	40.4 ± 15.8	47.3 ± 21.0	0.162
Duration (seconds)	27.9 ± 16.0	29.9 ± 22.5	0.549

Values are expressed as mean ± standard deviation or numbers.

*∗*Statistical significance; paired t-test.

## Data Availability

The data used to support the findings of this study are included within the article.
